# Effects of Using Rosemary Residues as a Cereal Substitute in Concentrate on Vitamin E, Antioxidant Activity, Color, Lipid Oxidation, and Fatty Acid Profile of Barbarine Lamb Meat

**DOI:** 10.3390/ani11072100

**Published:** 2021-07-15

**Authors:** Samir Smeti, Yathreb Yagoubi, Houssemeddine Srihi, Sandra Lobón, Juan Ramón Bertolín, Mokhtar Mahouachi, Margalida Joy, Naziha Atti

**Affiliations:** 1 Laboratoire de Productions Animales et Fourragères, INRA-Tunisia, University of Carthage, rue Hédi Karray, 2049 Ariana, Tunisia; yagoubiyathreb@hotmail.fr (Y.Y.); houssemsrihi60@gmail.com (H.S.); naziha.atti@gmail.com (N.A.); 2 Centro de Investigación y Tecnología Agroalimentaria de Aragón (CITA), Instituto Agroalimentario de Aragón-IA2, CITA-Universidad de Zaragoza, Avda, Montañana, 50059 Zaragoza, Spain; slobon@cita-aragon.es (S.L.); jrbertolin@cita-aragon.es (J.R.B.); mjoy@cita-aragon.es (M.J.); 3 Laboratoire Appui à la Durabilité des Systèmes de Production Agricole dans la Région du Nord-Ouest, ESAK, Le Kef, University of Jendouba, 7100 Jendouba, Tunisia; taymallahmah@gmail.com

**Keywords:** Barbarine lambs, meat quality, by-products, faba bean, soybean, polyphenols

## Abstract

**Simple Summary:**

The aim of this study was to investigate the effects of the inclusion of rosemary residues (RR) and protein sources on lamb meat quality. Twenty-four male Barbarine lambs were divided into three homogeneous groups receiving individually: 600 g of oat hay as a roughage supplemented with 600 g of commercial concentrate for the control group (C), concentrate containing rosemary residues (RR) plus soybean meal for the RRS group, and RR plus faba bean for the RRF group. The inclusion of RR did not affect meat lipid oxidation, but improved meat nutritional properties by increasing its polyphenol and polyunsaturated FA content. The source of crude protein did not affect any parameter studied.

**Abstract:**

The shortage of some ingredients and, consequently, the continuous increase in the price of feed encourage the search for other alternatives to maintain animal production and enhance its products. In this line, the use of aromatic plant by-products in animal diet has been recently and widely considered, given their richness in bioactive compounds. Therefore, the aim of this study was to investigate the effects of the inclusion of rosemary residues (RR) and protein sources on lamb meat quality. The experiment was carried out on 24 male Barbarine lambs (3 months old) with an average body weight (BW) of 17.8 ± 2.6 kg, which were divided into three homogeneous groups according to BW. The diet comprised 600 g of oat hay and 600 g of concentrate. Three types of concentrate were evaluated: commercial concentrate as the control group (C); rosemary residues (RR) plus soybean meal as the RRS group, and RR plus faba bean as the RRF group. After an experimental period of 65 days, lambs were slaughtered. The inclusion of RR in both concentrates increased the α-tocopherol and total polyphenol content in meat and protected meat against discoloration (high red index and chroma after 9 days of storage) but did not affect meat lipid oxidation, which was similar for all groups. The FA profile was affected by the inclusion of RR, with no effect from the source of protein (faba bean or soybean). The inclusion of RR in the concentrate increased the C18:2 n-6, C18:3 n-3, C20:4 n-6, C20:5 n-3, and C22:5 n-3 content (*p* < 0.05). Consequently, the inclusion of RR also increased the total polyunsaturated FA (*p* < 0.05) and the ratio of polyunsaturated FA to saturated FA (*p* < 0.05). The results of this study demonstrate that concentrate based on RR could be useful for lamb meat production by improving the nutritional quality of meat, especially the fatty acid profile. In addition, soybean meal can be replaced by faba bean in lamb concentrate without affecting meat quality.

## 1. Introduction

An increasing preference for natural foods has encouraged the food industry to include natural antioxidants in many products, instead of synthetic antioxidants, to retard lipid oxidation and improve the quality and nutritional value of some foods [1,2]. There is recent interest in nutritional strategies to improve animal product quality, primarily with the goal of producing natural, safe, and healthy meat or milk. In this context, the extracts and essential oils from aromatic or medicinal plants (rosemary, myrtle, sage, and thyme, among others) are rich in compounds with significant antioxidant activity [3]. This antioxidant capacity promotes a considerable interest in their use as alternative ingredients in animal diets [3,4,5,6,7]. In fact, improvement of meat quality is related to richness in polyphenols and vitamins, known for their antioxidant activity and used in order to reduce meat oxidation and extend the shelf life of meat [8,9].

Among the aromatic and medicinal plants (AMP) of Tunisia, rosemary (*Rosmarinus officinalis* L.) covers vast areas of the country. The industrial extraction of this plant generates a great amount of residues (5460 tons/year) [10], which could be valorized as alternative feed for sheep [4,11]. Many studies used rosemary residues or essential oil as additives to the basal diet of young lambs or ewes [4,11]. However, to the best of our knowledge, only one study dealt with their use as fodder that totally substituted the basal diet [6], and none studied their introduction in concentrate. Moreover, the use of local protein sources promotes the replacement of soybean in order to reduce dependency [12]. Soybean agriculture faces new challenges: on one hand, the increase in food and feed needs for humans and animals due to population growth, and on the other, the demand for agricultural production that guarantees health security, nutritional quality, and environmental sustainability. Therefore, this study aimed to evaluate the effects of the dietary inclusion of rosemary residues associated with two types of protein sources (soybean or faba bean) on meat quality of Barbarine lambs.

## 2. Materials and Methods

All procedures employed in this study (transport and slaughtering) meet ethical guidelines and adhere to Tunisian legal requirements (The Livestock Law No. 2005-95 of 18 October 2005, Chapter II; Section 1 and Section 2 relative to the slaughter of animals).

### 2.1. Animals, Diets, and Slaughter Procedure

The experiment was carried out at the experimental farm of the High School of Agriculture of Kef (ESAK) using 24 male Barbarine lambs (3 months old) weighing, on average, 17.8 ± 2.6 kg body weight (BW). Further details on the experimental design and slaughtering procedures were reported previously by [13]. Briefly, the lambs were divided into 3 homogeneous groups of 8 lambs each. Animals were fed twice a day and had free access to fresh water. Diet was individually controlled and comprised 600 g of oat hay and 600 g of concentrate. Each group received a different concentrate: commercial concentrate (control group, C); rosemary residues (RR) plus 600 g faba bean (RRF group); and RR plus soybean meal (RRS group). The ingredients and chemical composition of the concentrate are presented in Table 1.

After 65 days, lambs were slaughtered with an average BW of 25.7, 24.5, and 25.1 kg for C, RRF, and RRS, respectively [13]. After slaughter, carcasses were chilled at 4 °C for 24 h and then split longitudinally; the whole *longissimus thoracis and lumborum* muscle (LTL) from both sides was removed and sampled in further meat quality analyses. The LTL muscle was sliced and packed to determine the chemical composition, the α-tocopherol, retinol, cholesterol, and total phenolic content, and the FA profile. The LTL muscles from the 6th to the 13th thoracic vertebrae were sliced into 2.5-cm-thick samples and randomly assigned to 4 display times (0, 3, 6, and 9 d), placed in trays, wrapped with oxygen-permeable polyvinyl chloride film, and kept in darkness at 4 °C until measurement for color and lipid oxidation. The 0-d samples were also allowed to bloom in darkness at 4 °C for 1 h before measurement. All samples were immediately vacuum-packed and frozen (−20 °C) until further analysis.

### 2.2. Vitamin E, Retinol, and Cholesterol Analysis

Vitamin E (tocopherol) analysis in feedstuffs was performed according to the methodology proposed by [14]. Tocopherols were extracted 3 times with 3 mL of methanol-acetone-petroleum ether (1:1:1, 0.01% BHT). Next, 1 mL of the supernatant was evaporated in a vacuum evaporator. The dry residues were dissolved in 1 mL of acetonitrile: dichloromethane: methanol (75:10:15) and filtered through a 0.22-µm PTFE filter into a 2-mL glass screw-top vial and the determination was performed with the procedure described by Chauveau-Duriot et al. [15].

The vitamin E, retinol, and cholesterol content of the meat were determined following the procedure of [16]. Briefly, 200 mg of lyophilized sample was saponified with 3 mL of saponification solution (10% *w/v* potassium hydroxide in ethanol: distilled water 50:50 *v*:*v* mixture) overnight in inert N_2_ atmosphere. Then, the analytes were extracted twice with 5 mL of n-hexane: ethyl acetate (9:1, *v*:*v*) with 5 μg mL^−1^ of BHT mixture and evaporated in a vacuum evaporator. The dry residue was dissolved in 1 mL of acetonitrile: dichloromethane: methanol (75:10:15) and transferred into a 2-mL glass screw-top vial after filtering through a 0.22-µm PTFE filter.

In both analyses, the chromatographic system was an ACQUITY UPLC H-Class liquid chromatograph (Waters, Milford, MA, USA) equipped with a silica-based bonded phase column (Acquity UPLC HSS T3, 1.8 μm × 2.1 mm × 150 mm column, Waters), an absorbance detector (Acquity UPLC Photodiode Array PDA eλ Detector, Waters), and a fluorescence detector (2475 Multi λ Fluorescence Detector, Waters). The UPLC system was controlled using the Empower 3 software. Tocopherols were detected by fluorescence emission at 295 nm excitation wavelength (λexc) and 330 nm emission wavelength (λemi), retinol by absorbance at 325 nm, and cholesterol at 220 nm. Quantification was performed using a five-point calibration curve from pure standards.

### 2.3. Determination of Total Phenolic Content (TPC)

The total phenolic content (TPC) in feedstuffs were determined using the Folin–Ciocalteu method as described by Makkar et al. [17]. To this end, 200 mg of the sample was extracted twice with a 5-mL mixture of ultrapure water: acetone: formic acid (47.5:47.5:5, *v*:*v*:*v*). Subsequently, 20 µL of both extracts, 0.98 mL of ultrapure water, 0.5 mL of the Folin–Ciocalteu reagent, and 2.5 mL of 20% (*w/v*) Na_2_CO_3_ in ultrapure water were mixed in a 10-mL tube with a vortex stirrer for 35 min at room temperature. Finally, the absorbance was read at 725 nm using a Heλiosβ spectrophotometer (Thermo Electron Corporation, Waltham, MA, USA).

To determine the total phenolic content (TPC) in meat, the method of Vázquez-Vázquez et al. [18] was used with some modifications. Briefly, 1 g of lyophilized meat and 9 mL of milli-Q water (Ultramatic GR Wasserlab) were mixed in a 50-mL polypropylene tube and 10 mL of aqueous solution of methanol (50:50, *v*:*v*) was added. The obtained solution was vortexed for 5 min before 500 μL of Carrez I solution (Scharlau) and 500 μL of Carrez II solution were added and shaken for 1 min. Next, 5 mL of acetonitrile was added and shaken for 5 min. The samples were centrifuged at 4000 rpm for 15 min at 4 °C. The upper layer was filtered into a 15-mL polypropylene tube using a 0.22-μm polytetrafluoroethylene (PTFE) filter. Finally, 15 µL of extract, 147 µL of milli-Q water, 13 µL of Folin–Ciocalteu reagent, and 7% Na_2_CO_3_ were mixed in a microplate well and left to react for 30 min before the absorbance was read at 725 nm using an Epoch microplate spectrophotometer (BioTek, Winooski, VT, USA).

### 2.4. Determination of Antioxidant Activity

To determine antioxidant activity, the ABTS method was used [19]. Briefly, a 7 mM ABTS solution was prepared and mixed in a 1:1 ratio (*v*:*v*) with a 2.45 mM solution of K2S2O8, shaken in a vortex, and allowed to react overnight (approximately 16 h) to form ABTS. Subsequently, a solution of ABTS in absolute ethanol with an absorbance value of 0.700 ± 0.02 at 730 nm was prepared. Antioxidant activity extracts were taken and diluted 1/20 (*v*/*v*) for feedstuff and not diluted for meat—these extracts were the same as those used for the determination of polyphenols. Finally, 20 µL of the diluted extracts and 280 µL of ABTS solution were mixed to react for 30 min at room temperature (12 replicates of each extract were made). Once the reaction was complete, the absorbance at 730 nm was measured using the EPOCH microplate spectrophotometer (BioTek, Winooski, VT, USA).

### 2.5. Meat Color and Lipid Oxidation Analysis (TBARS)

Meat color of LTL samples was measured using a Minolta CM-2006 d chromameter (Konica Minolta Holdings, Osaka, Japan). The LTL samples were wrapped with oxygen-permeable PVC film, randomly assigned to 4 trays (0, 3, 6, and 9 days of storage), and kept in darkness at 4 °C. On each day of color measurement, samples were measured twice and averaged. Color coordinates (L*, a*, b*) were measured and C*, H* were calculated in the CIELAB space as H* = tan^−1^ (b*/a*) × 57.29, expressed in degrees, and C* = (a*^2^ + b*^2^) ^1/2^. After color measurement, the lipid oxidation (TBARS) assay was performed according to the method of Botsoglou et al. [20].

### 2.6. Fatty Acids

The fatty acid (FA) profile was determined via gas chromatography with a flame ionization detector for fatty acid methyl esters (FAMEs). The FA composition of feedstuffs (500 mg) was extracted as proposed by [21] using C19:0 as internal standard and the FA composition of meat samples (0.5 g of lyophilized and minced meat) was extracted according to Lee et al. [22] using C23:0 as internal standard. In both cases, FA were methylated with CH_3_ONa/CH_3_OH (0.5M) and acetyl chloride/CH_3_OH (1/10 *v*/*v*), extracted in heptane, and injected into a Bruker SCION 436-GC (Bruker, Billerica, MA, USA) equipped with a CP-8400 autosampler, a SP-2560 capillary column (200 m × 0.25 mm × 0.2 µm film thickness), and a flame ionization detector. FAs were identified using several commercial FA methyl ester standards (GLC-532, GLC-401, GLC-642, GLC-643, GLC-538, and GLC-463, Nu-Chek, Elysian, MN, USA) and a recommended bibliography [23,24,25]. FAMEs were quantified following the indications of UNE-EN ISO 12966-4:2015. Saturated (SFA), monounsaturated (MUFA), and polyunsaturated (PUFA) FAs were calculated. The desirable fatty acids were calculated as DFA =$\;\sum^{}$MUFA +$\;\sum^{}$PUFA + C18:0. The atherogenic (AI) and thrombogenic (TI) indexes were calculated according to Ulbricht and Southgate [26].

### 2.7. Sensory Evaluation

Meat samples were thawed at 4 °C, wrapped in aluminum foil, and placed in a preheated oven at 180 °C until the core temperature reached 71 °C [27]. Immediately after cooking, muscle was divided into 1 × 1 cm cubed samples, placed on white plastic trays, and individually marked with random digits for evaluation. Bread and mineral water were provided for the panelists to freshen their mouths between every two samples in a controlled room (the temperature was between 20 and 22 °C with 60–70% humidity). The sensorial panel was made up of trained, permanent researchers and remained consistent throughout the sensory analysis of meat samples, as recommended by AMSA [27]. There were 10 members and each panelist evaluated 8 samples per session over 3 sessions. They were asked to note the tenderness (1 = extremely tough, 9 = extremely tender), juiciness (1 = extremely dry, 9 = extremely juicy), flavor (1 = very poor, 9 = very good), and global acceptability (1 = not acceptable, 9 = extremely acceptable).

### 2.8. Statistical Analysis

Meat tocopherol, retinol, cholesterol, and phenolic compound content, antioxidant capacity, FA profile, and sensory quality data were analyzed using the GLM procedure of SAS [28]. A one-way ANOVA was used to test the effect of the substitution of standard concentrate with the two experimental concentrates based on rosemary distillation residues. The color and lipid oxidation of the LTL muscle were analyzed using the MIXED procedure of SAS for repeated measurements. The types of concentrate, the meat display time, and their interactions were included as fixed effects, and lamb was included as a random effect. The means were compared by the Duncan test. The significance was set at 5%.

## 3. Results and Discussion

### 3.1. Meat Vitamin E, Total Phenolic Compounds, Antioxidant Activity, Retinol, and Cholesterol Content

The meat from both RR groups (RRS and RRF) was found to contain twice the α-tocopherol content of the C group (Table 2; *p* < 0.001). This result is in agreement with Loetscher et al. [29], studying chicken, and Yagoubi et al. [6], studying lamb. The RRS and RRF concentrates presented a high α-tocopherol content, which is not completely degraded in the rumen but is deposited in muscle [30] where its antioxidant action is more effective. Similar results were observed when by-products of aromatic plants were fed to sheep [6,7]. The feeding treatment did not affect the δ-tocopherol, retinol, or cholesterol content in meat, in agreement with Yagoubi et al. [6], who totally replaced oat hay with RR in the diets of Barbarine lambs.

The inclusion of RR in both concentrates increased the phenolic compound content in meat compared with control (Table 2; *p* < 0.05), similar to the results observed when goats were fed with Moringa leaves [31]. The source of CP did not affect any of the chemical compounds studied (*p* > 0.05), except for ϒ-tocopherol (*p* < 0.05), which was found to be lower in concentrate with soybean meal.

### 3.2. Color and Lipid Oxidation (TBARS) Evolution

The meat color parameter evolution is presented in Table 3. The interaction between treatment and time was significant only for yellowness. The RRF presented lower values than control at day 3 (5.6 vs. 7.5) and RRS at day 6 (7.7 vs. 10.1), but presented similar values at day 0 (3.8) and day 9 (9.99). Feeding treatment did not affect the meat lightness (L*) and hue angle (H*) but did affect the redness (a*) and chroma (C*). These results are in line with other results which indicated that the inclusion of natural sources of phenolic compounds (olive cake, orange, and carob pulp) in the diet of beef cattle or lambs affected the meat color [32,33,34]. Meat from all treatments presented lightness values that were in the range of average acceptability [35]. Throughout the 9 days of storage, L* and C* were unaffected, whereas, a*, b*, and H* changed significantly (*p* < 0.05). A bright red color in lamb meat is desirable and attractive for consumers [36], however, during storage the redness values decreased gradually until the 9th day of storage, regardless of treatment. Redness is related to the level of antioxidant, such as vitamin E, which reduces myoglobin oxidation post-slaughter and consequently enhances color stability during storage [6,37]. The yellowness index increased throughout the 9 days of refrigerated storage at atmospheric conditions, similarly to the findings of Ripoll et al. [38] and de la Fuente-Vazquez et al. [39]. The chroma value was unchanged during the entirety of the storage period, contrary to the results previously reported by Yagoubi et al. [6] which demonstrated that the incorporation of aromatic plant leaves in sheep diets induced C* decrease as a result of meat pigment oxidation.

The inclusion of RR and the source of protein did not affect the lipid oxidation (*p* > 0.05). This lack of effect due to RR was unexpected as polyphenols have a powerful antioxidant activity which preserves meat from lipid oxidation [40,41]. The literature indicates an antioxidant activity in muscle when RR is included in the diet of broiler, turkeys, and ewes [42,43,44]. Moreover, the inclusion of RR in the diet of lambs showed a significant reduction in lipid oxidation [6]. This difference between studies could be due to the higher level of RR inclusion (60 and 87%) in the cited study compared with the current one (33%). Regarding the evolution, lipid oxidation increased with the time of display (*p* < 0.001). The TBARS values increased gradually and significantly from the 3rd day of storage until the 9th day to reach a value of 1.41 mg MDA/kg of meat. This value was higher than the acceptability threshold (1 mg MDA/kg of meat) reported by [45], but below the threshold of 2 mg MDA/kg of meat, as suggested by Campo et al. [46], for sensory detection of abnormal flavors and when meat is unacceptable to consumers.

### 3.3. Fatty Acid Profile

The intramuscular FA profile of lambs is presented in Table 4. The palmitic (C16:0), stearic (C18:0), and oleic (C18:1) acids were the most abundant FAs, representing more than 80% of the total detected FAs. The SFA total was not affected by the feeding treatment (*p* > 0.05) and the most abundant SFAs were palmitic (C16:0) and stearic (C18:0) acids [47,48]. The inclusion of RR tended to reduce the palmitic acid content (*p* = 0.065), whereas the C20:0 acid content increased compared with control (*p* < 0.05). The rest of the individual SFAs were not affected by feeding treatment. Regarding monounsaturated FAs, oleic acid was the most abundant which is in line with the values reported for intramuscular FAs of thin-tailed [49] and fat-tailed sheep [50,51]. The inclusion of RR did not affect the oleic acid content, also in line with previous studies in sheep and lamb [44,52].

The most abundant PUFA was linoleic acid (C18:2 n-6) and its content increased when RR was included in the diet (*p* < 0.05), likely due to its high RR by-product content (38 and 40%). In the same line, the inclusion of RR also increased the α-linolenic acid (C18:3n-3), C20:4 n-6, C20:5 n-3, and C22:5 n-3 content. Most of these FAs are associated with a positive effect on human health via the prevention of cardiovascular, metabolic, and inflammatory diseases and cancer [53]. The greater content of linoleic and linolenic acids in both RR treatments (*p* < 0.05) are in agreement with results observed by Yagoubi et al. [6].

From the point of view of human health, the nutritional quality of meat can be evaluated in terms of SFA, MUFA, PUFA, n-6, n-3 PUFA, and several important ratios [54]. These nutritional indicators are presented in Table 5. The SFA content was similar among treatments. In contrast, Yagoubi et al. [52] reported a reduction in SFA in the intramuscular fat of LTL due to the inclusion of RR as forage in lambs fed concentrate. This difference between studies could be due to the higher level of RR inclusion (60 and 87%) in the cited study than in the current one (33%). The total PUFA content increased with the inclusion of RR due to its partial escape from biohydrogenation in the rumen [9]. This result is in line with the tendency found by Yagoubi et al. [52]. Feeding treatment did not affect the ratio n-6: n-3, but affected the PUFA/SFA ratio and the thrombogenicity index (TI) (*p* < 0.05). The inclusion of RR in the concentrate increased the PUFA/SFA ratio and decreased the TI in line with nutritional recommendations.

No differences were found between RRF and RRS treatments in any individual FA or sum and principal ratios, and therefore the use of faba bean, a local resource, instead of soybean could be a good alternative to reduce dependency on soybean imports.

### 3.4. Sensory Evaluation

The results of RR incorporation on the sensory attributes of fresh meat are shown in Table 6. There was no significant difference among groups for all of the sensory attributes (*p* > 0.05). Meat from the different treatments was found to be moderately tender (score = 5.46), juicy (score = 4.96), odorous (score = 4.46), and tasty (score = 5.36), with a general acceptability score of 5.56. No panelist mentioned off flavors or odors for any group. The lack of dietary effects of rosemary by-products on sensory evaluation was previously reported for lamb meat [3,55]. The incorporation of RR did not have a negative effect on the meat. These results are in line with several previous findings showing that aromatic plant leaves, essential oils, and extracts could preserve meat eating quality by ameliorating its appearance, delaying its rancidity, and reducing off-flavor intensity [56,57,58].

## 4. Conclusions

The results demonstrate that the total substitution of the commercial concentrate with experimental concentrates based on distilled rosemary residues protected meat against discoloration. The inclusion of rosemary residues in the concentrate at up to 33% could be advisable as it improved meat nutritional properties by increasing its polyphenol and PUFA content with no negative effects on meat eating quality. Soybean meal can be totally replaced by faba bean as no effect was observed.

## Figures and Tables

**Table 1:** Centesimal and chemical composition of experimental feeds.

Item	Oat Hay	Standard Concentrate	RRF ^1^	RRS ^2^
Ingredients (%)				
Rosemary residues		0	33	31
Barley		20	22	39
Soybean meal		7.5	0	16
Wheat bran		37.5	8	11
Faba bean		0	29	0
Mineral vitamin supplement		4	3	3
Molasses		0	5	0
Salt		1	0	0
Corn		30	0	0
Chemical Composition				
Dry matter (%)	91.95	88.69	92.35	90.7
Crude protein (% DM)	5.47	16.33	17.30	17.38
Organic matter (% DM)	93.04	80.65	89.56	91.2
Neutral detergent fiber (% DM)	69.15	20.31	29.47	34.01
Acid detergent fiber (% DM)	40.94	10.17	18.52	19.05
Acid detergent lignin (% DM)	3.75	2.33	7.15	9.14
Total phenolic content (Tannic acid/g DM)	1.01	0.48	1.53	1.63
α-Tocopherol (µg/g DM)	4.42	0.45	52.71	62.98
γ-Tocopherol (µg/g DM)	3.97	0.78	11.85	7.4
Fatty Acid Profile (% of total FAMEs)				
C12:0	0.77	0.07	0.09	0.14
C14:0	1.46	0.42	0.54	0.50
C15:0	0.28	0.13	0.12	0.10
C16:0	45.73	34.72	28.54	27.87
C18:0	27.51	16.45	13.25	11.37
C18:1 9c	6.07	21.16	13.95	13.57
C18:1 11c	0.65	1.87	1.22	1.29
C18:2 n6	10.68	23.51	38.31	40.85
C18:3 n3	6.49	1.07	3.73	4.15

^1^ concentrate with rosemary residues and faba bean, ^2^ concentrate with rosemary residues and soybean meal.

**Table 2:** Tocopherols, retinol, cholesterol, antioxidant activity, and phenolic compounds of meat.

Item	C	RRF	RRS	SEM	*p*-Value
α-tocopherol μg/g FM	0.84 ^b^	1.62 ^a^	1.58 ^a^	0.122	0.001
ϒ-tocopherol ng/g FM	44.98 ^a^	38.48 ^a^	20.97 ^b^	4.592	0.004
δ-tocopherol ng/g FM	7.13	6.85	6.83	0.696	0.945
Retinol ng/g FM	33.00	33.78	30.78	4.521	0.878
Cholesterol mg/g FM	0.64	0.64	0.65	0.021	0.866
Total phenolic content ^1^	51.33 ^b^	60.34 ^a^	60.29 ^a^	2.110	0.008
Antioxidant activity (ABTS) ^2^	0.30	0.34	0.36	0.021	0.23

C: control group fed standard concentrate; RRF: experimental group fed concentrate with rosemary residues and faba bean; RRS: experimental group fed concentrate with rosemary residues and soybean meal; FM: fresh matter. The letters a and b within a row with different superscripts indicate significant differences among the feeding treatments (*p* < 0.05); ^1^ Expressed as [Tannin acid] (Tannic acid/g DM); ^2^ Expressed as [TROLOX] (µmol/g DM).

**Table 3:** Effect of the inclusion of residues of rosemary and time of storage on color and lipid oxidation (mg MDA/kg meat) evolution.

	Diets (D)			Time (T)				Statistics			
	C	RRF	RRS	0	3	6	9	RSD	D	T	D*T
L*	43.00	43.18	44.58	43.31	43.19	44.59	43.24	3.546	0.3733	0.4633	0.7063
a*	14.73 ^xy^	13.96 ^y^	15.41 ^x^	16.93 ^a^	15.17 ^b^	14.07 ^b^	12.63 ^c^	2.320	0.0484	<0.0001	0.058
b*	7.38	6.82	7.69	3.83 ^d^	6.55 ^c^	8.79 ^b^	9.99 ^a^	1.769	0.1626	<0.0001	0.0325
C*	17.01 ^xy^	16.02 ^y^	17.54 ^x^	17.42	16.62	16.90	16.49	2.410	0.0414	0.5525	0.1041
H*	27.42	26.92	25.53	12.53 ^d^	23.12 ^c^	32.18 ^b^	38.67 ^a^	5.039	0.3004	<0.0001	0.1695
**TBARS**	0.82	0.92	0.66	0.32 ^d^	0.55 ^c^	0.92 ^b^	1.41 ^a^	0.391	0.3875	<0.0001	0.4444

C: control group fed standard concentrate; RRF: experimental group fed rosemary pellets containing faba bean; RRS: experimental group fed rosemary pellets containing soybean. The letters a, b, c and d within a row with different superscripts indicate significant differences among the storage times (*p* < 0.05). The letters x and y within a row with different superscripts indicate significant differences among the feeding treatments.

**Table 4:** Effect of rosemary residue intake on fatty acid profile of Barbarine lamb meat (mg FA/100 g FA).

Item	C	RRF	RRS	SEM	*p*
SFA					
C10:0	0.31	0.31	0.29	0.032	0.911
C11:0	0.01	0.01	0.01	0.003	0.33
C12:0	0.53	0.45	0.50	0.052	0.565
C13:0	0.03	0.02	0.03	0.004	0.848
C14:0	4.57	3.87	4.25	0.379	0.444
C15:0	0.56	0.50	0.52	0.032	0.431
C16:0	26.11	23.88	24.24	0.702	0.065
C17:0	1.31	1.19	1.20	0.078	0.495
C18:0	16.55	16.34	16.22	0.544	0.909
C19:0	0.18	0.17	0.17	0.022	0.9
C20:0	0.09 ^b^	0.13 ^a^	0.13 ^a^	0.011	0.036
C21:0	0.01	0.01	0.01	0.004	0.827
C22:0	0.01	0.01	0.01	0.002	0.472
C24:0	0.006 ^b^	0.020 ^a^	0.010 ^b^	0.0031	0.017
DMA-C16:0	1.49	1.98	0.91	0.207	0.215
DMA-C18:0	0.617 ^b^	0.980 ^a^	0.951 ^a^	0.104	0.039
DMA-C18:1	0.592	0.537	0.496	0.028	0.079
MUFA					
C14:1	0.11	0.12	0.14	0.016	0.708
C15:1-9c	0.11	0.15	0.15	0.018	0.158
C16:1-9c	1.28	1.19	1.14	0.059	0.265
C17:1 9c	0.28	0.39	0.36	0.043	0.174
C18:1 9c	27.47	26.18	26.02	0.707	0.299
C20:1	0.08	0.08	0.09	0.010	0.639
C22:1	0.01	0.01	0.02	0.004	0.342
C24:1	0.01	0.01	0.01	0.004	0.95
C18:1 10t	0.67	0.63	0.55	0.155	0.87
C18:1 11t	1.82	1.54	1.70	0.105	0.18
PUFA					
C18:2 n6	3.58 ^b^	6.14 ^a^	5.66 ^a^	0.455	0.001
C18:2 9c,11t	0.81	0.65	0.75	0.051	0.111
C18:3 n6	0.03	0.05	0.05	0.007	0.225
C18:3 n3	0.688 ^b^	0.834 ^a^	0.880 ^a^	0.046	0.021
C19:2 n6	0.01	0.01	0.01	0.003	0.365
C20:2 n6	0.04	0.06	0.04	0.009	0.481
C20:3 n6	0.121 ^b^	0.237 ^a^	0.233 ^a^	0.025	0.005
C20:3 n3	0.01	0.03	0.01	0.007	0.185
C20:4 n6 ARA	0.994 ^b^	1.822 ^a^	1.590 ^a^	0.196	0.02
C20:5 n3 EPA	0.253 ^b^	0.460 ^a^	0.433 ^a^	0.055	0.029
C22:4 n6	0.05	0.09	0.08	0.019	0.3
C22:5 n6	0.016	0.044	0.020	0.008	0.058
C22:5 n3 DPA	0.326 ^b^	0.607 ^a^	0.561 ^a^	0.067	0.015
C22:6 n3 DHA	0.0626	0.151	0.120	0.026	0.071

SFA: saturated fatty acid; MUFA: monounsaturated fatty acid; PUFA: polyunsaturated fatty acid; C: control group fed standard concentrate; RRF: experimental group fed rosemary pellets containing faba bean; RRS: experimental group fed rosemary pellets containing soybean. The letters a and b within a row with different superscripts indicate significant differences among the feeding treatments (*p* < 0.05).

**Table 5:** Effect of the inclusion of rosemary residues on fatty acid groups (mg FA/100 g FA).

Item	C	RRF	RRS	SEM	*p*
SFA	54.95	52.40	53.18	0.930	0.163
MUFA	36.40	34.68	34.56	0.606	0.079
PUFA	8.64 ^b^	12.9 ^a^	12.25 ^a^	0.798	0.002
ƩBranched Chain FA	1.86	1.84	1.97	0.088	0.53
Σ n6	4.83 ^b^	8.44 ^a^	7.68 ^a^	0.655	0.002
Σ n3	1.339 ^b^	2.078 ^a^	2.010 ^a^	0.155	0.004
n6/n3	3.58	4.07	3.83	0.196	0.222
PUFA/SFA	0.159 ^b^	0.248 ^a^	0.231 ^a^	0.018	0.004
ƩConjugated linoleic acid	1.00	0.87	0.99	0.060	0.274
18:1 10t/C18:1 11t	0.37	0.42	0.35	0.100	0.872
Atherogenicity index	1.07	0.89	0.95	0.066	0.187
Thrombogenicity index	1.91 ^a^	1.59 ^b^	1.64 ^b^	0.085	0.031

SFA: saturated fatty acid; MUFA: monounsaturated fatty acid; PUFA: polyunsaturated fatty acid; CLA: conjugated linoleic acid; C: control group fed standard concentrate; RRF: experimental group fed rosemary pellets containing faba bean; RRS: experimental group fed rosemary pellets containing soybean. The letters a and b within a row with different superscripts indicate significantly differ among the feeding treatments (*p* < 0.05).

**Table 6:** Effect of rosemary residue intake on the sensory attributes of Barbarine lamb meat.

Sensorial Parameters	C	RRF	RRS	SEM	*p*-Value
Odor	4.4	4.6	4.4	0.15	0.54
Tenderness	5.8	5.2	5.4	0.27	0.29
Juiciness	5.1	5.0	4.8	0.22	0.64
Flavor	5.4	5.3	5.4	0.12	0.95
General acceptability	5.5	5.5	5.6	0.14	0.88

C: control group fed standard concentrate; RRF: experimental group fed rosemary pellets containing faba bean; RRS: experimental group fed rosemary pellets containing soybean.

## Data Availability

The data presented in this study are available on request from the corresponding author.
